# Advances in Monitoring Cell-Based Therapies with Magnetic Resonance Imaging: Future Perspectives

**DOI:** 10.3390/ijms18010198

**Published:** 2017-01-19

**Authors:** Ethel J. Ngen, Dmitri Artemov

**Affiliations:** 1 In Vivo Cellular and Molecular Imaging Center, Division of Cancer Imaging Research, Russell H. Morgan Department of Radiology and Radiological Sciences, Johns Hopkins University School of Medicine, Baltimore, MD 21205, USA; dartemo2@jhmi.edu; 2 Sidney Kimmel Comprehensive Cancer Center, Johns Hopkins University School of Medicine, Baltimore, MD 21205, USA

**Keywords:** cell-based therapies, cell-tracking, cellular MRI, MRI contrast agents, environmentally-responsive MRI biosensors

## Abstract

Cell-based therapies are currently being developed for applications in both regenerative medicine and in oncology. Preclinical, translational, and clinical research on cell-based therapies will benefit tremendously from novel imaging approaches that enable the effective monitoring of the delivery, survival, migration, biodistribution, and integration of transplanted cells. Magnetic resonance imaging (MRI) offers several advantages over other imaging modalities for elucidating the fate of transplanted cells both preclinically and clinically. These advantages include the ability to image transplanted cells longitudinally at high spatial resolution without exposure to ionizing radiation, and the possibility to co-register anatomical structures with molecular processes and functional changes. However, since cellular MRI is still in its infancy, it currently faces a number of challenges, which provide avenues for future research and development. In this review, we describe the basic principle of cell-tracking with MRI; explain the different approaches currently used to monitor cell-based therapies; describe currently available MRI contrast generation mechanisms and strategies for monitoring transplanted cells; discuss some of the challenges in tracking transplanted cells; and suggest future research directions.

## 1. Introduction

Cell-based therapies are currently being developed and evaluated for applications in both regenerative medicine and in oncology [1,2,3]. There are currently 14,831 completed, and 8325 open on-going clinical trials on cell-based therapies throughout the world, registered on the United States National Institute of Health (NIH) clinical trials website [4,5]. Of these trials, ~68% of the completed trials (10,034) and ~71% of the open trials (5896) are related to cancer treatment [6,7].

**Figure 1:**
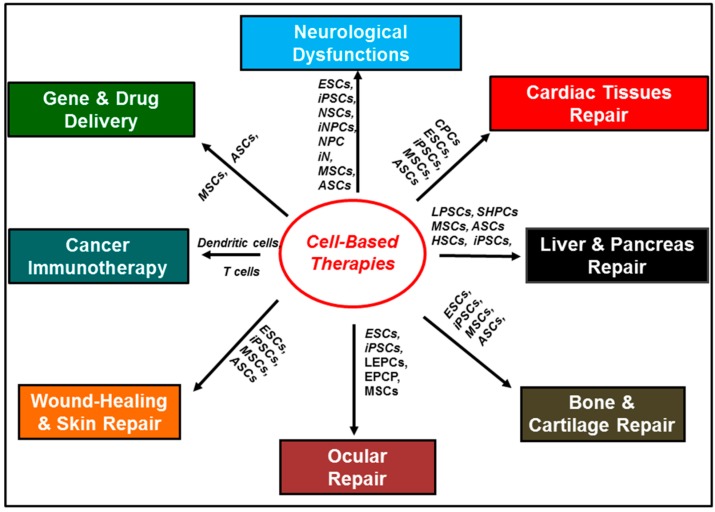
Schematic representing applications for cell-based therapies in regenerative medicine and in oncology. The following cell types are abbreviated in the figure: embryonic stem cells (ESCs); neural stem cells (NSCs); neural progenitor cells (NPCs); mesenchymal stem cells (MSCs); induced pluripotent stem cells (iPSC); induced neuronal cells (iN); induced neuronal progenitor cells (iNPCs); adipose-derived stem cells (ADSCs); embryonic germinal stem cells (EGC); endothelial progenitor cells (EPCs); cardiac progenitor cells (CPCs); lens epithelial progenitor cells (LEPCs); epithelial progenitor cells (EPCP); small hepatocytes-like progenitor cells (SHPCs); liver stem cells/progenitor cells (LPSCs); sinusoidal endothelial progenitor cells (SEPCs); hematopoietic stem cells (HSCs); and adipose stem cells (ASCs).

In regenerative medicine, stem cell therapies enable the repair of damaged tissue either directly, by replacing injured cells in the tissue of interest, or indirectly, by paracrine signaling at the injury site, which stimulates the repair process [8,9,10]. Given the limited regenerative ability of the central nervous system (CNS), stem cell therapies are currently being investigated as potential solutions to a wide range of CNS-related disorders and injuries [11,12,13]. Applications for cell-based therapies in CNS regenerative medicine include: The reversal of neurodegeneration associated with diseases such as Alzheimer’s disease, Parkinson’s disease, amyotrophic lateral sclerosis (ALS), Huntington’s disease, and demyelinating disorders, such as multiple sclerosis (MS) [12,14,15,16,17,18,19,20,21,22]. The reversal of the neurological deficits associated with spinal cord injuries, stroke, traumatic brain injuries, and brain tumor therapy-related injuries, such as radiotherapy-induced brain injuries [23,24,25,26,27,28,29,30,31,32,33,34,35]. Stem cell therapies are also being investigated for wound-healing, and for the repair of damage to a variety of tissues, including cardiac, ocular, liver, bone, and cartilage tissue (Figure 1) [36,37,38,39,40,41,42,43,44,45,46,47,48,49,50,51].

A variety of stem cells have been explored for both cell replacement therapies and to modulate physiological responses through paracrine action. Table 1 gives examples of the different types of stem cells that have been tested for various pathologies. Given the large number of preclinical studies that have been carried out, this table is not exhaustive but rather provides an overview.

In oncology, immune cells, such as dendritic cells, and natural and engineered T cells are being explored for cancer immunotherapy [52,53,54,55,56,57]. Given the migratory properties of stem cells in response to chemokines secreted in the tumor-microenvironment, stem cells such as mesenchymal stem cells, capable of phagocytosing therapeutic loads, are currently being explored as delivery vehicles for drugs, genes, and imaging agents [58,59,60]. Cell-based therapies are also currently used in the treatment of hematological malignancies, such as leukemia [61,62].

While cell-based therapies present potential solutions to a variety of problems in regenerative medicine and in oncology, preclinical research on these cell-based therapies and their translation to the clinic will benefit tremendously from imaging approaches that enable the noninvasive monitoring of the delivery, survival, migration, distribution, and integration of transplanted cells. This will permit the noninvasive assessment of the fate of transplanted cells longitudinally without the need for invasive biopsies and histological assessment, and enable the tailoring and personalization of cell-based therapeutic regimens.

## 2. Current Trends in Cellular Imaging

**Figure 2:**
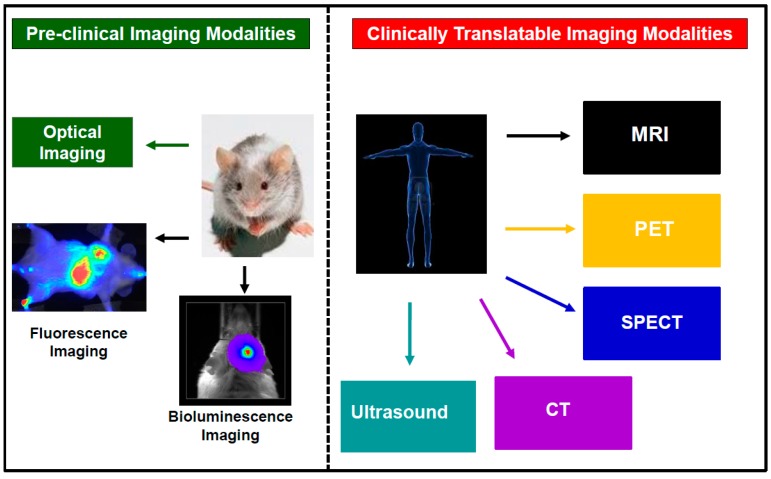
Schematic representing the different imaging modalities used in tracking cell-based therapies both preclinically and clinically. The following imaging modalities are abbreviated in the figure above: magnetic resonance imaging (MRI), positron emission tomography (PET); single photon emission computed tomography (SPECT); and computed tomography.

Several imaging modalities have been used to track transplanted cells both preclinically in small animal models and clinically in humans [63,64]. These include: optical imaging (fluorescence and bioluminescence imaging); nuclear imaging (positron emission tomography (PET) and single photon emission computed tomography (SPECT)); computed tomography; ultrasound imaging; and magnetic resonance imaging (MRI) (Figure 2). These modalities all have advantages, but also limitations for tracking transplanted cells. These advantages and limitations have been well documented in several recent review articles [63,64].

In this review, we focus on MRI as a tool for imaging transplanted cells. MRI has several advantages over other imaging modalities for tracking transplanted cells. A major advantage of MRI is that, unlike nuclear imaging which uses unstable radioactive isotopes as probes, with short lifetimes that generate ionizing radiation, MRI probes are not generated from radioactive isotopes, hence are stable and do not generate ionizing radiation. This permits the serial and longitudinal assessment of transplanted cells, at high spatial resolution without exposure to ionizing radiation [63,64]. Although, MRI is generally several orders of magnitude less sensitive than optical and nuclear imaging (detection limits of approximately 10^−3^–10^−5^ M for MRI versus 10^−9^–10^−17^ M for optical imaging and 10^−10^–10^−12^ M for nuclear imaging) [63,64], it is possible to image single cells labeled with superparamagnetic iron oxide nanoparticles (SPIONs) in clinical 3T scanners, due to the “blooming” or magnetic susceptibility artifact, which causes the signal from the particles to extend beyond the immediate surroundings of the contrast agent, as a result of magnetic field inhomogeneities [65,66,67]. Thus, in combination with its high spatial resolution, MRI might be better suited than optical and nuclear imaging for tracking transplanted stem cells. There is currently a need for the development of MRI probes that permit the visualization of specific cellular and molecular processes.

## 3. The Principle of Cell Tracking with MRI

**Figure 3:**
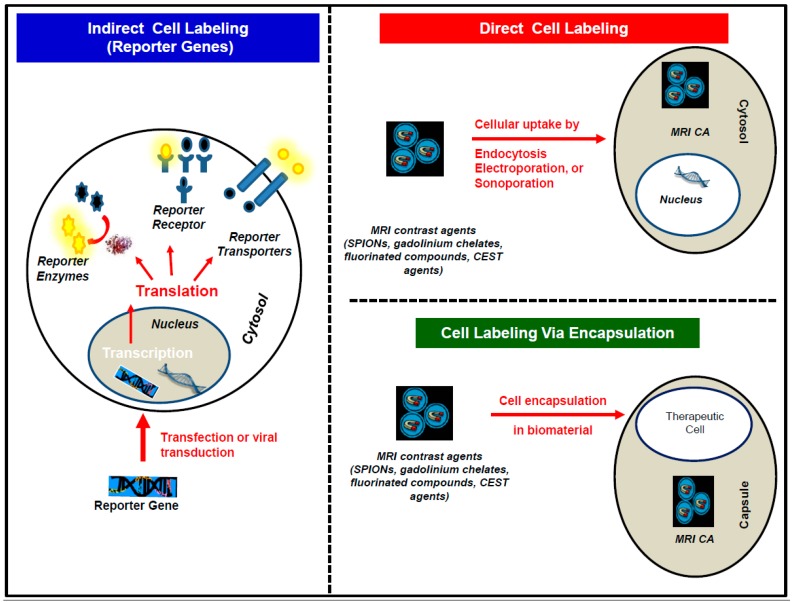
Schematic representing the different cell labeling approaches. Where, CA stands for contrast agents; CEST stands for chemical exchange saturation transfer; and SPIONs stands for superparamagnetic iron oxide nanoparticles.

In order to track the delivery, migration, and survival of transplanted cells with MRI, it is imperative to endow the cells with MRI-sensitive properties via cell labeling, so that the cells can be detected after transplantation. Currently, three cell labeling techniques are generally used to endow cells with these properties: The direct cell labeling technique; the indirect cell labeling technique; and the encapsulation cell labeling technique (Figure 3).

### 3.1. The Direct Cell Labeling Technique

In the direct cell labeling technique, cells are incubated with an MRI contrast agent in vitro, and, prior to transplantation, usually with a transfection agent, such as poly-l-lysine (PLL) or lipofectamine [68,69,70]. The contrast agent is then endocytosed via either pinocytosis or phagocytosis, depending on its size. Other methods, such as electroporation and sonoporation have also been used to directly label cells with MRI contrast agents [71,72,73].

Although, the in vitro direct cell labeling technique is most often used to label cells prior to transplantation, several groups have demonstrated the feasibility of directly labeling endogenous cell populations in vivo, by targeting either the phagocytic nature of endogenous cell populations; or by targeting their cell surface receptors with either ligand or antibody-conjugated MRI contrast agents [74,75,76,77,78,79]. However, this in vivo direct cell labeling technique is usually used for either pathological diagnosis or to elucidate the role and the mechanism of action of endogenous cell populations in various pathologies [80,81]. Recently, in vivo labeling of cells prior to cell harvest and transplantation has been suggested [82,83]. However, this method is not generally used yet, to track transplanted cells. Several limitations of the in vivo cell labeling method have been identified. These include the following: This method will be limited to the labeling and harvesting of cells which are phagocytic in nature and easy to isolate, such as bone marrow-derived MSCs. For example, although it has been demonstrated that neural stem cells can be directly labeled in vivo, the difficulty of isolating these cells from the subventricular zone precludes the clinical application of this method for labeling and harvesting neural stem cells. This in vivo labeling approach will be most useful for allogeneic transplants, since in autologous transplants the patient would also have labeled phagocytic macrophages that would be difficult to distinguish from the transplanted MSCs. Additionally, since not all donors yield sufficient labeled stem cells for clinical dosing, the in vivo labeled cells harvested will have to be expanded prior to transplantation, and this could result in the serial dilution of the MRI signal from the labeled cells to uncertain detection levels. More immune-phenotyping studies will also be required to ensure that the labeled cells harvested are indeed MSCs and not macrophages. Finally, this method does not provide a means to distinguish live transplanted cells from dead cells and could result in false-positive signals in the case of cell death, macrophage influx and secondary particle uptake. However, this method will still be clinically useful for tracking labeled cell transplantation in real-time using MR-compatible catheters and also for monitoring cell engraftment [83].

The exogenous direct cell labeling technique is currently the most employed cell labeling method, due to its simplicity and ease of use. However, it has a number of limitations. These include: The inability to effectively distinguish live labeled cells from dead labeled cells [84,85]. Since most MRI contrast agents such as SPIONs, paramagnetic gadolinium chelates, manganese-based nanoparticles, and perfluorocarbon nanoemulsions, all generate an MRI contrast whether in solution, within transplanted cells, or upon transfer of the contrast agent to infiltrating phagocytic immune cells such as macrophages, during graft rejection, it is usually difficult to distinguish live labeled cells from dead labeled cells [86,87]. Although several groups have suggested potential solutions for some MRI contrast generation mechanisms, this is still an area of active research and translational studies are still needed to standardize these proposed methods [88,89,90,91]. Examples of some of the proposed solutions for distinguishing live labeled cells from dead labeled cells include exploiting the differences in the transverse and longitudinal relaxation rates of T_2_ and T_1_ contrast agents respectively, when compartmentalized in live cell organelles compared to the relaxation rates of the free contrast agents when released from lysed dead cells [89,90,92]. Another proposed solution has been to exploit the effects of pH changes which usually accompany cell death, on the chemical exchange saturation transfer (CEST) rates of CEST contrast agents, to distinguish live cells from dead cells [93,94]. These examples and mechanisms are described in detail in Section 4 below. Another limitation of the exogenous direct cell labeling technique is the inability to reliably, serially quantify the proliferation and migration of labeled transplanted cells, due to the dilution of the MRI signal as the cells proliferate. Since a fixed amount of the contrast agent is present within the labeled cells after direct cell labeling, the MRI signal from the labeled transplanted cells decreases over time, as the contrast agent is distributed among the daughter cells during cell proliferation (contrast dilution) [90,95]. This renders precise cell quantification difficult.

### 3.2. The Indirect Cell Labeling Technique

In the indirect cell labeling technique, cells are either transiently transfected with the help of transfection agents or transduced with viral vectors to express an MRI reporter gene. An MRI reporter gene is a gene that can be either fused to a gene of interest or cloned instead of a gene of interest. Upon the expression of the MRI reporter gene as a peptide, protein nanostructure, receptor, enzyme, or cellular transporter it can either generate an inherent MRI contrast or interact via receptor binding, enzymatic activation or cellular efflux, with an administered MRI contrast agent to generate an MRI contrast.

Although, the indirect cell labeling technique is more complex than the direct cell labeling technique, it has several advantages over the direct cell labeling technique [63,64]. For example: Since the indirect cell labeling technique involves genetically engineering the cells to express the reporter gene of interest, the engineered cells proliferate to generate daughter cells that express the reporter gene of interest. Consequently, the MRI signal is not diluted as the cells proliferate [63,64]. Furthermore, live labeled cells can be reliably distinguished from dead labeled cells [86,87]. Since the labeled cells are genetically engineered to express the reporter gene of interest, the gene is expressed only in live cells and switched off in dead cells. This makes it possible to distinguish live cells from dead cells and accurately determine the survival of transplanted cells [63,64]. Recently, a study was carried out to evaluate the effectiveness of the direct cell labeling method using superparamagnetic iron oxide nanoparticles (SPIONs) compared to the indirect cell labeling method using genetically overexpressed ferritin (an iron storage protein). Briefly, mouse skeletal myoblasts were either labeled with SPIONs or genetically engineered to overexpress ferritin. Along with the two live labeled cell transplant mouse groups, two other mouse groups received dead labeled cell transplants. In the two mice groups which received SPIONs labeled cells, live cells could not be distinguished from dead cells. However, in cells labeled with ferritin, only live cells were detected. Although ferritin was successful in distinguishing live cells from dead cells, the signal obtained from the ferritin labeled live cells was much lower compared to that obtained from the SPIONs labeled live cells [86].

Several other MRI reporter gene systems which use the different MRI contrast generation mechanisms have been developed [96,97]. Examples of MRI reporter gene systems that can be detected with the T_2_/T_2_* MRI contrast generation mechanisms include: the iron storage protein ferritin described above [98,99]; and the iron-binding receptor transferrin, responsible for cellular iron internalization [100]. However, these receptors and proteins all require the administration of exogenous T_2_/T_2_* MRI contrast agents such as SPIONs. The enzymes β-galactosidase which catalyzes the hydrolysis of β-d-galactosides is an example of a reporter gene that can be detected with the T_1_ MRI contrast generation mechanism [101]. However, this system also requires the administration of exogenous T_1_ contrast agents such as gadolinium chelates. Most recently, several reporter gene systems which use the CEST contrast generation mechanism and do not require the administration of exogenous contrast agents have also been developed. These include: the lysine rich protein (LRP); the super charged green fluorescent protein (ScGFP); human protamine-1 (hPRM-1) and the protein kinase A (PKA) sensor [102,103,104,105]. Other CEST MRI reporters which require the administration of exogenous CEST contrast agents have also been developed. These include the enzymes: herpes simplex virus type-1 thymidine kinase (HSV1-TK); and cytosine deaminase (CD) [106,107].

However, since the indirect cell labeling technique is still in its relative early stages of development, it still faces a few limitations that call for future research [97]. For example: In cases where an interaction with an MRI probe is needed to generate a signal, the use of imaging agents with unfavorable pharmacokinetic profiles could lead to a delayed MRI signal and consequently result in false-negative reporting. Additionally, since extremely high transduction efficiencies could impair the normal biological functioning of the engineered cells, transducing cells with optimal efficiencies to express the reporter gene of interest while still retaining the normal biological function could lead to low detection sensitivities, especially given the inherently low sensitivity of the currently available probes. Finally, a limitation that is often overlooked is that, since most of the currently developed genetically encoded reporters are of non-human origins (usually of bacterial origins), they are therefore immunogenic. While this might not pose a problem for the short-term (5–10 days) tracking of transplanted cells, the use of immunogenic reporters will limit their usefulness for longitudinal cell fate studies in the clinical setting. Thus, highly sensitive and non-immunogenic reporter genes and specific MRI probes with suitable pharmacokinetic profiles are needed for MRI.

### 3.3. The Encapsulation Cell Labeling Technique

Although the encapsulation technique has traditionally not been considered one of the cell labeling techniques, it has been included in this review due to its growing use. In the encapsulation cell labeling technique, biomaterials such as alginate capsules are used to protect therapeutic cells from infiltrating immune cells. Alginate is a biocompatible polymer, purified from algae, that has been extensively used for cell encapsulation [108,109]. These alginate capsules permit the diffusion of low molecular weight compounds, thus are permeable to small molecules such as water and nutrients but impermeable to infiltrating immune cells. Polycations such as poly-l-lysine (PLL) have been used extensively to control the pore sizes (permeability) of the capsules and also to provide stability for the capsules [108,109]. Generally, these capsules are approximately 350 µm in diameter and can hold varying numbers of single cells depending on the cell size [110]. For human islet cells, one islet is generally used per capsule [109]. However, for single cells such as MSCs, up to 300 MSCs can be enclosed in a single capsules, and up to 500 capsules can be administered [108,109]. Both the therapeutic cells and the MRI contrast agents are encapsulated together in vitro, prior to transplantation [108,109,110,111,112]. The MRI contrast agent then generates a signal that reports on the status of the transplanted cells.

While this method can be used to report on the delivery and survival of transplanted cells, it is not designed to report on cell migration or integration.

## 4. MRI Contrast Generation Mechanisms

Several MRI contrast agents that function through different contrast generation mechanisms have been developed and used to track transplanted cells. These include: Agents that affect the transverse relaxation rates (R_2_/R_2_*) of water protons in their surroundings (T_2_/T_2_* agents); agents that affect the longitudinal relaxation rates (R_1_) of water protons in their surroundings (T_1_ agents); agents with exchangeable protons or coordinated water molecules that can be saturated with specific radiofrequency pulses, and that can transfer the saturation to surrounding non-saturated water protons or molecules via chemical exchange saturation transfer (CEST agents); and agents that possesses NMR-detectable nuclei not typically found in biological systems, such as fluorine (^19^F), which can generate MRI “hot spots”. These agents all have advantages for tracking transplanted cells, but also limitations, which provide avenues for future research and development.

### 4.1. T_2_/T_2_* Contrast Agents

T_2_/T_2_* contrast agents are currently the most widely used MRI contrast agents for tracking transplanted cells both preclinically and clinically [113,114,115]. T_2_/T_2_* agents function by decreasing the transverse relaxation rates (R_2_/R_2_*) of water protons in their surroundings, predominantly via perturbation of the magnetic field homogeneity, and have a lesser effect on the longitudinal relaxation rates (R_1_) of water protons [116,117]. Since the perturbation of the magnetic field homogeneity by these agents results in the loss of the MRI water signal, their presence is identified on MR images by a signal void or darkening (hypointensity) of their local surroundings [116,117]. However, this signal void could also result from other sources such as chemical shift artifacts, hemorrhage, and air bubbles and could lead to a misinterpretation of the images. This false-positive misinterpretation is generally not encountered with the other MRI contrast agents which generate positive contrast such as T_1_ agents and fluorine “hot spot” agents [118].

Although the most widely used T_2_/T_2_* MRI contrast agents in preclinical studies and in clinical trials are superparamagnetic iron oxide nanoparticles (SPIONs) [119,120], there are currently no T_2_/T_2_* MRI contrast agents FDA-approved for tracking transplanted cells. However, clinical grade SPIONs, FDA-approved for other applications have been employed in several cell-tracking clinical trials [113]. Clinical grade SPIONs, FDA-approved for liver imaging that have been used in clinical trials for tracking transplanted cells include: an SPION with a dextran coating called Endorem^®^ in Europe and Feridex^®^ in the USA; and an SPION with a carboxydextran coating called Resovist^®^ [113,121,122]. However, the production of Feridex^®^ was discontinued in 2009, due to commercial considerations, since there was little demand for its FDA-approved application [113]. Feromuxytol, an ultra-small superparamagnetic iron oxide nanoparticle (USPION), FDA-approved for the treatment of iron deficiencies in patients with renal failure [123], has also been suggested for tracking stem cells [124]. However, USPIONs (~5 nm in diameter) are generally less sensitive than SPIONs (~80–150 nm in diameter) for tracking transplanted cells. Thus, developing clinical grade SPIO-based MRI contrast agents for tracking transplanted cells is an area of active research. This includes developing agents with large SPIO cores and biocompatible polymer surfaces, such as poly lactic-co-glycolic acid (PLGA), an FDA-approved polymer for drug delivery [125,126,127].

There is also a need for the development of nanoparticles with different degradation kinetics and subsequently different lifetimes, which could be used for different cell tracking purposes. For example, since immune cells have short lifespans, monitoring immune cells will be better achieved with particles with faster degradation kinetics. This would prevent the persistence of the particles long after the immune cells have died, and prevent the detection of false-positive signals [128]. Imaging stem cells, which have much longer lifespans, would however, require particles with longer lifetimes. In a particle degradation kinetics study, it was demonstrated that the rate of particle degradation was affected primarily by the particle surface coating and secondarily by the particle size [128]. The rate of particle degradation was faster in particles coated with more biodegradable polymers such as PLGA than in particles coated with cellulose. In PLGA particles, the rate of degradation was faster in PLGA-coated nanoparticles than in PLGA-coated microparticles. This is expected, since PLGA nanoparticles were initially designed for drug delivery and degradation of the particles to release the drug load was key to the success of these particles [126]. While the degradation of these particles and their ability to track MSCs has been demonstrated in vivo [125], the use of these particles to track transplanted immune cells in vivo short-term still needs to be demonstrated.

T_2_/T_2_* MRI agents function predominantly by perturbing the magnetic field homogeneity in their local surroundings, and this perturbation is greater with particles of larger iron core sizes that possess larger magnetic moments [66,129]. Thus, several strategies have been developed, based on this principle to improve the sensitivity of T_2_/T_2_* agents. These include developing micron-sized particles that possess larger SPIO cores [130,131]. Genetically encoded reporters have also been developed, which produce iron-binding proteins such as ferritin, an iron storage protein, which can bind to iron endogenously present in the organism and thus increase the intracellular iron concentration or bind to administered iron oxide nanoparticles, to form MRI-sensitive large iron aggregates [99,132,133,134]. This strategy was used to distinguish live mouse skeletal myoblasts genetically engineered to express ferritin from dead genetically engineered cells transplanted to the mouse heart [86].

While labeling cells with T_2_/T_2_*-genetically encoded reporters can be used to track cell delivery, migration, survival and differentiation, SPIO-based T_2_/T_2_* MRI agents are most effective in tracking cell delivery and migration, but are difficult to use in tracking cell survival and differentiation [86,87,135]. Thus, several strategies for predicting cell survival with SPIO-based T_2_/T_2_* MRI agents have been developed [88,89,90,92,95,136,137,138]. These strategies exploit molecular and cellular differences between live and dead cells in modulating changes in the relaxivity. These molecular and cellular differences include differences in: cell membrane permeability, enzymatic activity, pH, and proliferation rates [139,140].

**Figure 4:**
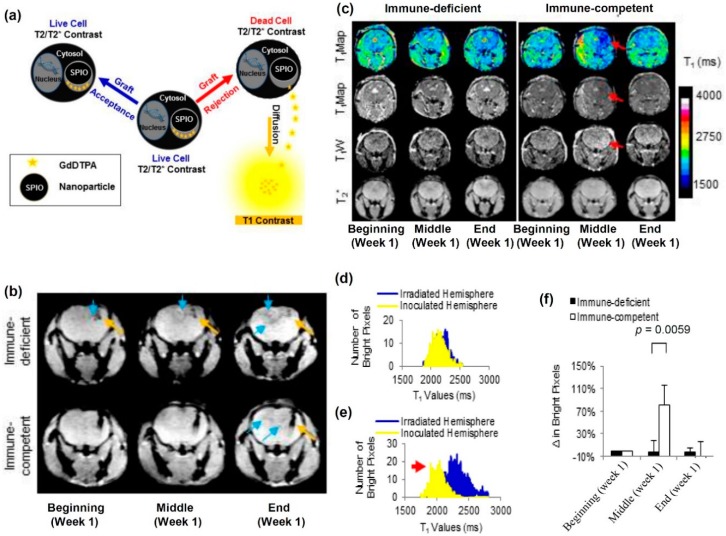
Cell tracking using the MRI dual contrast technique. (**a**) Schematic representing live cell-tracking by T_2_/T_2_* contrast enhancement, and cell death detection by T_1_ contrast enhancement. Where SPIO stands for superparamagnetic iron oxide and GdDTPA stands for gadolinium-diethylenetriaminepentaacetic acid. (**b**) T_2_*-weighted images of immune-deficient and immune-competent mouse brains, indicating the site of cell delivery (↑) and cell migration to the radiation-induced lesion (↑). More cell migration to the injury site was detected in immune-deficient mice; (**c**) Comparison of T_1_ contrast enhancement in immune-deficient and immune-competent mice, respectively, within the first week of cell transplantation. A significant T_1_ contrast enhancement was observed in the slice adjacent to that of the cell delivery site in immune-competent mice. (↑) represents T_1_ contrast enhancement in the slice adjacent to that of cell delivery; (**d**) Pixel intensity histograms of the ipsilateral and contralateral hemispheres of cell implantation before graſt rejection indicate similar T_1_ values in both hemispheres; (**e**) Pixel intensity histograms of the ipsilateral and contralateral hemispheres of cell implantation aſter graſt rejection indicate lower T_1_ values in the hemispheres ipsilateral to cell implantation. (↑) represents T_1_ contrast enhancement in the slice adjacent to that of cell delivery; (**f**) Quantification of T_1_ contrast enhancement in immune-deficient and immune-competent mice, respectively, at the beginning, middle, and end of week one. The signals were normalized for each mouse and indicate significant T_1_ contrast enhancement within week one in immune-competent mice, indicating cell death. The images and caption are reprinted from Ngen et al. [88].

A particularly promising, yet simple approach called the MRI dual contrast technique was recently developed to detect cell death of transplanted SPION-labeled cells in real time [88]. This MRI dual contrast method involves labeling cells with both a high molecular weight (low diffusion coefficient) T_2_/T_2_* agent such as SPIONs and a low molecular weight (high diffusion coefficient) T_1_ agent, such as gadolinium-based chelates [88,141,142]. In live cells, where the cell membrane is intact and both contrast agents are in close proximity to each other, the T_2_/T_2_* signal from the SPIONs predominate and mask the T_1_ signal from the T_1_ agent (Figure 4). This T_2_/T_2_* signal from the SPIONs can then be used to track cell delivery and migration (Figure 4b). However, in the case where the cells die after transplantation (in immune-competent mice), the cell membrane is disrupted, and both contrast agents are released from the dead cells. The T_1_ agent with a high diffusion coefficient diffuses away from the SPION with a low diffusion coefficient and generates a T_1_ signal, in the vicinity of the T_2_/T_2_* signal (Figure 4c–f). This T_1_ signal is then used to indicate cell death. Both the T_2_/T_2_* signal and the T_1_ signal can be separated using a spin echo pulse sequence and appropriate acquisition parameters, when both contrast agents are as little as ~15 µm away from each other [88,141,142]. This dual contrast cell labeling technique was used to track MSCs transplanted to repair radiation-induced brain injury (RIBI) in a mouse model (Figure 4). However, the dual contrast cell labeling technique could also be applied to monitor other types of stem cells such as NSCs, transplanted to repair traumatic brain injury or even stroke, clinically.

While this environmentally-responsive SPIO-based T_2_/T_2_* nanosystem and others proposed are promising, this is still an area of active research. These nanosystems and acquisition methods still need to be optimized and validated before they can be clinically translated. For example, given the rapid clearance of low molecular weight gadolinium chelates after they are released from dual labeled dead cells, the imaging schedule is very important in obtaining accurate readings. Thus, for this method to be clinically translated, a more standardized imaging schedule will need to be defined. Additionally, given the intrinsically lower sensitivity of MRI to detect low molecular weight gadolinium chelates compared to SPIONs, this method is currently most suitable for the detection of hyper acute and acute cell death, where high concentrations of the gadolinium chelates are released instantaneously from a large number of dead cells, and can be detected with MRI (detection limit within the micromolar concentration range). Thus, for this nanosystem to be applied universally for detecting cell death, gadolinium chelates with slower clearance rates, compatible with standardized imaging schedules will be needed. Finally, since this method relies on the diffusion of low molecular weight gadolinium chelates through breached cell membranes to detect cell death, and also since low molecular weight gadolinium chelates (<800 Da) are sufficiently small (<2 nm in diameter) to diffuse through apoptotic pores generated during apoptosis, this method is not designed to distinguish between apoptotic versus necrotic cell death mechanisms. For this method to detect specific cell death mechanisms, probes based on the dual contrast technique and capable of sensing biomarkers associated with specific cell death mechanisms such as enzymatic expression, will be needed.

### 4.2. T_1_ Contrast Agents

T_1_ contrast agents function by reducing the longitudinal relaxation rate (R_1_) of water protons in the surroundings of the agents. This leads to a gain in the MRI signal and results in a brightening of the voxels with high concentrations of the agents on T_1_-weighted MR images [116,117].

Traditionally, T_1_ MRI contrast agents that have been used to track transplanted cells have been paramagnetic gadolinium-based and manganese-based agents [143,144,145]. However, T_1_ MRI contrast agents are less often used in tracking cell-based therapies than T_2_/T_2_* agents, due to their lower sensitivity. Several nanotechnology strategies have thus been developed to overcome this limitation. These include developing nanoparticles and liposomes with large clusters of the paramagnetic agents to enhance the sensitivity of the agents [146,147,148,149]. However, T_1_ agents require direct contact with the surrounding water protons to modulate the contrast as opposed to T_2_/T_2_*, which do not require direct contact with the surrounding water protons, but can disrupt the local magnetic field homogeneity in the vicinity of the magnetic nanoparticle and modulate the contrast [116,117]. Thus, mesoporous nanoparticles (usually silica-based nanoparticles doped with the paramagnetic agents) that enable the direct contact of the paramagnetic agents in the nanoparticles with surrounding water protons have been developed [143,145,146].

Another challenge in using T_1_ MRI contrast agents is the possibility of quenching the T_1_ relaxation, depending on the cellular localization and concentration of the agent. In cases where high concentrations of the agents are sequestered in the lysosomes of the cells, with limited water accessibility, this could lead to a quenching of the T_1_ relaxation [150,151]. Thus, cell labeling techniques that enable the cytosolic localization of the agents at appropriate concentrations, such as electroporation and sonoporation, have also been investigated [150,151].

Although, gadolinium-based and manganese-based T_1_ contrast agents have been used to monitor cell delivery and migration; monitoring cell survival and differentiation has been a challenge [63,64]. Several strategies that modulate the relaxivity by exploiting the molecular and cellular differences between live and dead cells have been developed [90,152,153]. This is an ongoing area of research [140,154]. An example which exploits the differences in the cell membrane permeability between live and dead cells in modulating changes in longitudinal relaxation rates (R_1_) has been used to distinguish live cells labeled with gadolinium chelates from dead labeled cells [90]. It was shown that the differences in the longitudinal relaxation rates of gadolinium chelates in live transplanted cells, where the agents are entrapped by the cell membrane and have limited water accessibility, differ from that of gadolinium chelates in dead cells, were the cell membranes are disrupted and the agents have more water accessibility.

**Figure 5:**
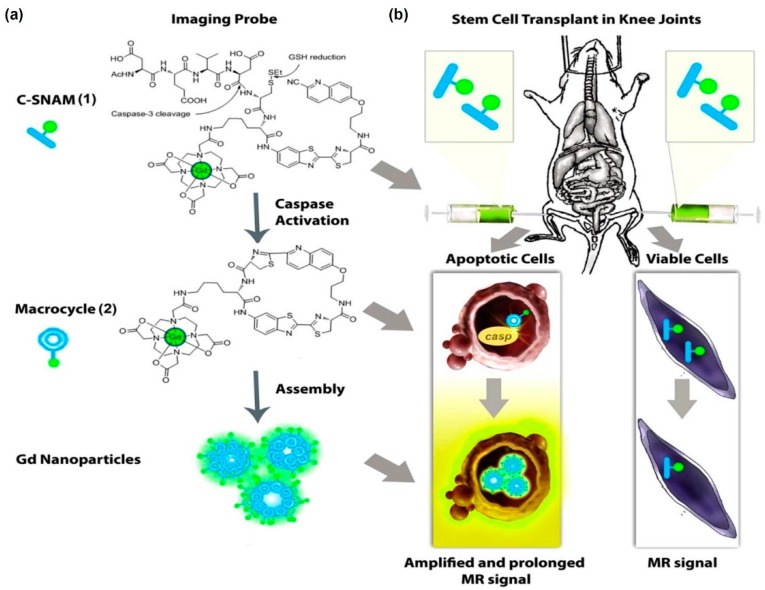
General design and mechanism of action of the caspase-3-sensitive nanoaggregation MRI probe (C-SNAM). (**a**) Chemical structure of C-SNAM. Following disulfide reduction and caspase-3-triggered DEVD peptide cleavage, C-SNAM transforms to a rigid and hydrophobic macrocyclic product 2, through a biocompatible intramolecular cyclization reaction between 2-cyano-6-hydroxyquinoline and d-cysteine residue. The macrocycle 2 will subsequently self-assemble into Gd nanoparticles, leading to an increase in longitudinal relaxivity (r_1_) relative to the unactivated probe 1; (**b**) Corresponding mechanism of action in vivo. (1) Intra-articular injection of C-SNAM into rat knee joints with implants of apoptotic and viable stem cells. (2) In vivo activation of C-SNAM in apoptotic stem cell transplants through caspase-3-mediated activation. (3) Increased relaxivity and retention effect of GdNPs lead to enhanced MRI signal of apoptotic stem cell transplants. The images and caption are reprinted with permission from Nejadnik et al. [152].

Another interesting approach was recently developed which exploits the expression of the caspase-3 enzyme (an apoptosis biomarker), to distinguish live cells from apoptotic cells, using a gadolinium-based caspase-3 activable probe (Figure 5) [152]. In this method called caspase-3-sensitive nanoaggregation MRI (C-SNAM), the probe self-assembles into nanoparticles after hydrolysis by caspase-3, released from apoptotic cells. This aggregation leads to an enhancement of the relaxivity and prolongs in vivo retention of the probe. Although, this method does not involve labeling the cells with the probe prior to transplantation, but rather depends on administering the probe locally at the transplantation site, it is a good example of tracking cell survival using environmentally-responsive T_1_ agents.

Several reporter gene systems that could be used to monitor gene expression in transplanted cells using activable T_1_ contrast agents have also been developed, such as the *lacZ* gene which encodes the enzymes β-galactosidase that catalyzes the hydrolysis of β-d-galactosides [101,155,156]. However, the extensive use of these systems has been limited due to their low sensitivity in vivo. Given the nephrotoxicity associated with gadolinium-based contrast agents, several non-metallic biosensors based on the chemical exchange saturation transfer contrast mechanism and fluorine MRI, described in Section 4.3 and Section 4.4 below, are currently being explored as alternatives [157,158,159].

### 4.3. Chemical Exchange Saturation Transfer (CEST) Contrast Agents

CEST contrast agents are a relatively new class of MRI contrast agents. These agents generate an MRI contrast by reducing the signal from water protons in their surroundings, following chemical exchange and saturation transfer from protons on the contrast agent or water molecules coordinated to the contrast agent and selectively saturated with an appropriate radiofrequency pulse, to water protons or free water molecules in their surroundings [160].

There are two main classes of CEST contrast agents: diamagnetic and paramagnetic CEST agents [161]. Generally, diamagnetic CEST (DIACEST) contrast agents are organic molecules with exchangeable protons such as amine, amide, and hydroxyl protons that can undergo chemical exchange and saturation transfer with the surrounding water protons, following selective saturation of the protons of interest. Since these agents are not metal-based, the toxicity associated with metal-based MRI contrast agents is avoided with their usage [159].

Paramagnetic CEST contrast agents (PARACEST), however, are usually chelates of paramagnetic lanthanide ions (metal-based). These agents generate contrast by reducing the signal from water protons in their surroundings, following the chemical exchange and saturation transfer of selectively saturated water molecules coordinated (bound) to the contrast agents with non-coordinated (unbound) free water molecules. PARACEST agents generate less background signal than DIACEST agents, due to the large chemical shift difference between the saturated coordinated water molecules of interest and the free water molecules. Both types of agents have been used to monitor transplanted cells [93,162]. Recently, PARACEST agents (europium and ytterbium chelates) were used to monitor tissue engineering by NSCs and endothelial cells within a stroke cavity in a preclinical rodent stroke model. The distribution of the different cell types within the lesion cavity and the individual contribution of the different cell types to morphogenesis were successfully monitored simultaneously using both PARACEST agents. This study demonstrated the importance of imaging agents to guide the delivery of the different cellular building blocks for de novo tissue engineering and to understand the dynamics of cellular interactions in de novo tissue formation [162].

**Figure 6:**
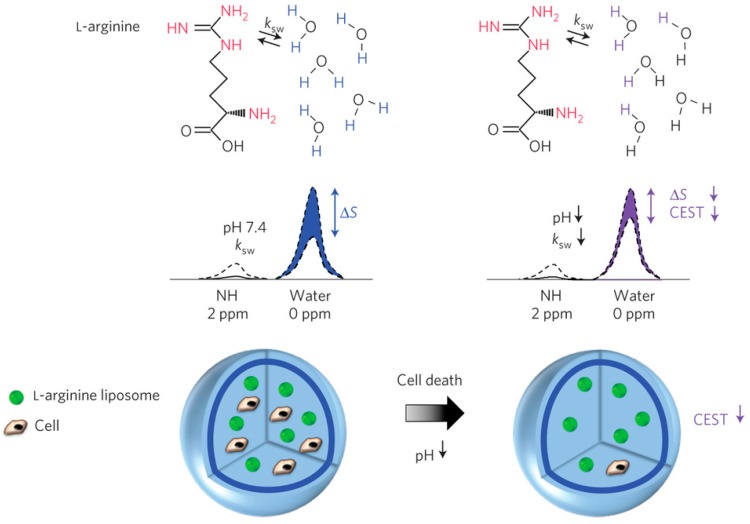
Schematic representing the principles of in vivo detection of cell viability using LipoCEST microcapsules as pH nanosensors. The CEST contrast is measured by the drop in the signal intensity (Δ*S*) of water after selective saturation (that is, removal of capability to generate signal) of the NH protons in l-arginine at 2 ppm. The l-arginine protons (red) inside the LipoCEST capsules exchange saturation (ksw) with the surrounding water protons. The ksw is reduced at lower pH causing a significant drop in CEST contrast. The images are reprinted with permission from Chan et al. [93].

Given the sensitivity of chemical exchange rates and chemical shifts to environmental factors such as pH and ionic strength and content, which are in turn affected by cell physiological conditions, CEST agents have been used as environmentally-responsive MRI biosensors to monitor cell viability [129,139]. An l-arginine liposome with multiple exchangeable amine protons was developed as a pH-sensitive DIACEST nanosensor to monitor cell death of encapsulated cells in vivo (Figure 6) [93]. This method exploits the sensitivity of the exchange rate of the guanidyl protons of l-arginine to pH changes in the range typically associated with the cell death process (pH 7.4–6.0). In live cells, where the pH is close to 7.4, the exchange rate between the saturated guanidyl protons of the l-arginine liposome and those of the surrounding bulk water protons is optimal. However, in apoptotic cells where the pH drops from pH 7.4 to about pH 6.0, the exchange rate decreases and subsequently the CEST signal also decreases. This decrease in the CEST contrast is then used to indicate cell death.

Genetically encoded CEST reporters have also been developed, which could enable the monitoring of transplanted cells [102,103,104,106]. Although these systems have been tested for cancer detection, their application in regenerative medicine still needs to be demonstrated. Examples of CEST genetically-encoded reporters include CEST-responsive peptides such as: the lysine rich protein (LRP); the super charged green fluorescent protein (ScGFP); human protamine-1 (hPRM-1) and the protein kinase A (PKA) sensor, which do not require the administration of exogenous contrast agents, [102,103,104,105]. Other CEST MRI reporters which require the administration of exogenous CEST contrast agents have also been developed. These include the enzymes: herpes simplex virus type-1 thymidine kinase (HSV1-TK); and cytosine deaminase (CD) [106,107]. However, the main limitation of these agents has been their relatively lower sensitivity compared to T_2_/T_2_* agents. This could affect the detection of transplanted cells in regenerative medicine where generally fewer cells per voxel need to be detected compared to tumor masses. Thus, developing more sensitive, non-immunogenic, genetically encoded reporters is an active area of research [163].

A third class of CEST contrast agents (hyperCEST agents), have very recently been developed. These agents exploit transfer between protein-bound hyperpolarized xenon (^129^Xe) and unbound hyperpolarized ^129^Xe [163,164]. The hyperCEST technique makes use of xenon-binding structures, such as cryptophanes, which induce large chemical shifts of ^129^Xe, between bound and unbound states [165,166,167]. Since these agents involve the use of hyperpolarized nuclei, they are more sensitive than other MRI agents [165,168]. However, the short lifetime of hyperpolarized ^129^Xe in vivo limits their extensive application.

### 4.4. Fluorine (^19^F) Contrast Agents

The fluorine-19 isotope (^19^F) is a stable NMR-detectable nuclei, which can be used in MRI, unlike the radioactive fluorine-18 isotope (^18^F) used in PET. ^19^F MRI has been used to track the delivery and migration of transplanted cells [70,169,170,171]. Since fluorine is not naturally found in biological systems, there is no background signal from the tissue when these agents are used. Given the relatively low sensitivity of ^19^F MRI, most systems used to track transplanted cells have been based on nanocarriers, such as perfluorocarbon-based (PFC) and perfluoropolyether (PFPE) nanoemulsions, which can hold several ^19^F atoms [172,173]. Dendritic cells which are being explored as immunotherapies for cancer and autoimmune diseases have been successfully labeled with PFPE. An intracellular concentration of 5.2 × 10^12^ fluorine spins or 0.25 ng of PFPE was determined by ^19^F NMR. The cells were then injected to mice either intravenously into tissue and monitored with ^19^F MRI, using an 11.7 T preclinical animal scanner. The anatomical location of the cells was determined by proton (^1^H) MRI.

There has been great enthusiasm for the use of PFC nanoemulsions in clinic due to their relatively low toxicity, their exceptionally high cell specificity and the possibility of cell quantification in vivo. However, one of the main challenges to the clinical translation of ^19^F MRI for tracking immune cells is the development of appropriate hardware sufficiently robust to image large areas of the human body [174,175]. Current methods to image immune cells rely on acquiring two sets of images: ^19^F MRI which gives information on the transplanted cells and ^1^H MRI which gives anatomical information that helps locate the cells. However, the image co-registration between the acquisitioning of the two nuclei images could vary due to the coil handling and/or subject motion. Secondly, the sensitivity of the images acquired from the different nuclei could differ. Thus, several efforts for the clinical translation of ^19^F MRI are focused on the development of dual radiofrequency (RF) coils capable of simultaneously acquiring ^1^H and ^19^F MRI in large subjects.

**Figure 7:**
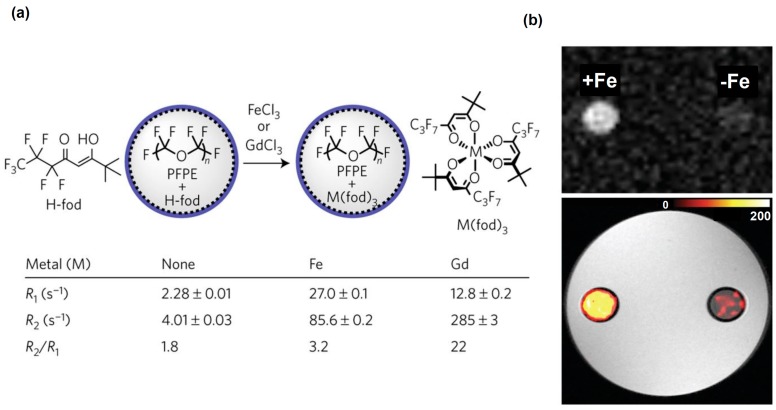
(**a**) Comparison of iron and gadolinium diketonates (H-fod) as ^19^F relaxation agents for PFPE. The relaxometry results (9.4 T) are shown for PFPE emulsions (120 g l^−1^ PFPE) containing H-fod (2.8 mM) 24 h after the addition of 0.7 mM metal ions. R_1_, spin–lattice relaxation rate (=1/T_1_), and R_2_, spin–spin relaxation rate (=1/T_2_), values are reported for the main PFPE peak at −91.4 ppm. The results show that Fe^3+^ is a more effective R_1_ agent than Gd^3+^. (**b**) MRI of FETRIS nanoemulsion. Phantom comprised of two agarose-embedded NMR tubes containing FETRIS nanoemulsion (4.5 g·L^−1^ ^19^F) with 0.5 mM Fe^3+^ (R_1_/R_2_ = 32.5/170 s^−1^) and nanoemulsion without metal (R_1_/R_2_ = 2.2/3.7 s^−1^), denoted +Fe and −Fe, respectively. The top panel shows unthresholded ^19^F images, and below, the ^19^F image is thresholded, rendered in hot-iron pseudo-color (scale bar), and overlaid onto the greyscale ^1^H image. The ^19^F/^1^H MRI data were acquired using a gradient echo (GRE) sequence. The images and caption are reprinted with permission from Kislukhin et al. [173].

Recently, formulations of PFPE nanoemulsions with improved sensitivity for cellular MR were also developed [173]. These constructs consisted of metal-binding β-diketones conjugated to linear PFPE. These fluorinated ligands were formulated as aqueous nanoemulsions and then metallated with various transition and lanthanide ions in the fluorous phase (Figure 7a). The iron (III) tris-β-diketonate (FETRIS) nanoemulsions, showed superior MRI properties and low cytotoxicity. The resulting ^19^F MRI signal was enhanced by three-to-five-fold over previously used tracers at 11.7 T, (Figure 7).

While ^19^F MRI has been used to track cell delivery and migration, tracking the functional status (survival and differentiation) of transplanted cells with these ^19^F nanosystems has been a challenge. Thus, future directions in this field will focus on developing environmentally-responsive ^19^F nanosystems capable of reporting on the functional status of transplanted cells. Several ^19^F systems, capable of sensing the presence of metal ions in their micro-environment such as zinc and iron, have been developed [176,177,178]. Since metal ions, such as zinc and iron ions, play a fundamental role in the functioning of cells, these systems could be developed to report on the functional status of transplanted cells. However, more research in preclinical models is still needed on these systems before they can be translated clinically.

Genetically encoded ^19^F reporters capable of reporting on the activity of transplanted cells have also been developed [179,180,181]. The feasibility of monitoring the expression and activity of β-galactosidase, the product of the *lacZ* gene, in transfected cells was demonstrated using ^19^F NMR chemical shift imaging (CSI), using different prototype reporter molecules [179,180,181]. However, like other reporter gene systems, for these systems to be translated to clinic, the regulatory hurdles associated with genetic engineering still need to be addressed. Additionally, the hardware limitations associated with imaging large subjects, discussed above, also need to be addressed.

## 5. Conclusions

Although cellular MRI is still in its infancy, several promising cellular MRI techniques have been developed to monitor the delivery, migration, and biodistribution of the transplanted cells. However, monitoring the functional states of transplanted cells, including their survival and differentiation, is still a challenge. Thus, future research in cellular MRI is bound to focus on the development and translation of environmentally-responsive MRI contrast agents, capable of reporting on the status of transplanted cells. Several strategies that exploit molecular and cellular differences between live and dead cells in modulating changes in relaxivity and chemical exchange rates are currently being explored for the development of environmentally-responsive MRI agents. These differences include differences in cell membrane permeability, enzymatic activity, and pH. The clinical translation of cell-based therapies would benefit tremendously from the development of more robust and sensitive probes with better pharmacokinetic profiles, which will permit the effective detection of specific cellular processes associated with cell death and differentiation at high spatial and temporal resolutions. This could accelerate the clinical translation and personalization of cell-based therapies.

## Figures and Tables

**Table 1:** Examples of the different types of cells evaluated for various pathologies.

Disease Type	Examples of Cells Tested	Cell Therapy Rationale	References
**Neurological Dysfunctions**			
Parkinson’s disease	Embryonic stem cells (ESCs); neural stem cells (NSCs); neural progenitor cells (NPCs); mesenchymal stem cells (MSCs); induced pluripotent stem cells (iPSC); induced neuronal cells (iN); induced neuronal progenitor cells (iNPCs).	Cell replacement therapy; immunomodulatory and neuroprotective properties	[11,12,13,14,15,16,17]
Alzheimer’s disease	ESCs; NSCs; NPCs; MSCs; iPSCs; iN; iNPCs	Cell replacement therapy; immunomodulatory and neuroprotective properties	[12,18,19,20]
Huntington’s disease	ESCs; NSC; NPC; MSC; adipose-derived stem cells (ADSCs).	Cell replacement therapy; immunomodulatory and neuroprotective properties	[12,13,14]
Amyotrophic lateral sclerosis	ESCs; NSCs; iPSCs; embryonic germinal stem cells (EGC)	Cell replacement therapy; immunomodulatory and neuroprotective properties	[12]
Multiple sclerosis	ESCs; iPSCs; MSCs; ADSCs;	Cell replacement therapy; immunomodulatory and neuroprotective properties	[20,21]
**Central and Peripheral Nervous System (CNS and PNS) Injuries**			
Spinal cord injuries	ESCs; MSCs; adipose-derived mesenchymal stem cells	Cell replacement therapy; neuroprotective properties.	[22,23,24]
Stroke	MSCs; ESCs; NSCs; iPSCs	Cell replacement therapy; immunomodulatory and neuroprotective properties.	[25,26,27]
Traumatic brain injuries	MSCs; iPSCs; bone-marrow-derived multipotent adult progenitor cells (MAPCs)	Cell replacement therapy; immunomodulatory and neuroprotective properties.	[28,29,30]
Radiotherapy-induced brain injuries	NSCs; ESCs; MSCs	Cell replacement therapy; immunomodulatory and neuroprotective properties.	[31,32,33,34,35]
**Tissue Repair**			
Skin (wound healing)	MSCs; ASCs; iPSCs; hematopoietic stem cells (HSCs); endothelial progenitor cells (EPCs)	Cell replacement therapy; paracrine action; modulation of physiological responses.	[36,37]
Heart	Cardiac progenitor cells (CPCs); MSCs; ASCs; iPSCs	Cell replacement therapy; paracrine action; modulation of physiological responses.	[38,39,40]
Eyes	Lens epithelial progenitor cells (LEPCs); epithelial progenitor cells (EPCP); inducible progenitor cells (iPSCs); MSCs.	Cell replacement therapy; paracrine action; modulation of physiological responses.	[41,42,43,44]
Liver	Small hepatocytes-like progenitor cells (SHPCs); Liver stem cells/progenitor cells LPSCs; Sinosoidal endothelial progenitor cells (SEPCs); Hematopoeitic Stem cells (HSCs); MSCs.	Cell replacement therapy; paracrine action; modulation of physiological responses.	[45,46]
Bone and cartilage	MSCs; ASCs.	Cell replacement therapy; paracrine action; modulation of physiological responses.	[47,48,49,50,51]
**Cancer Immunotherapy**			
Cancer	Dendritic cells; T cells	Stimulate immune response.	[52,53,54,55,56,57]
**Drug and Gene Delivery**			
Cancer	MSCs; ASCs.	Migratory properties.	[58,59,60]

## Abbreviations

ADSCs	Adipose-derived stem cells
ALS	Amyotrophic lateral sclerosis
CEST	Chemical exchange saturation transfer
CNS	Central nervous system
CPCs	Cardiac progenitor cells
CT	Computed tomography
DIACEST	Diamagnetic chemical exchange saturation transfer
EGCs	Embryonic germinal cells
EPCs	Epithelial progenitor cells
ESCs	Embryonic stem cells
FDA	Food and drug administration
^18^F	Fluorine-18 isotope
^19^F	Fluorine-19 isotope
HSCs	Hematopoietic stem cells
HyperCEST	Hyperpolarized chemical exchange saturation transfer
iN	Induced neuronal cells
iNPCs	Induced neuronal progeneitor cells
iPSCs	Induced pluripotent stem cells
LEPCs	Lens epithelial progenitor cells
LPSCs	Liver stem cells/progenitor cells
MRI	Magnetic Resonance Imaging
MS	Multiple sclerosis
MSCs	Mesenchymal stem cells
NIH	National Institute of Health
NPCs	Neural progenitor cells
NSCs	Neural stem cells
PARACEST	Paramagnetic chemical exchange saturation transfer
PET	Positron emission tomography
pH	Hydrogen potential
PLGA	Poly lactic-co-glycolic acid
PLL	Poly-l-lysine
R_1_	Longitudinal relaxation rate
R_2_	Transverse relaxation rate
SEPCs	Sinosoidal endothelial progenitor cells
SHPCs	Small hepatocytes-like progenitor cells
SPECT	Single photon emission computed tomography
SPIO	Superparamagnetic iron oxide
T_1_	Longitudinal relaxation time
T_2_	Transverse relaxation time
